# Evaluation of selected IL6/STAT3 pathway molecules and miRNA expression in chronic obstructive pulmonary disease

**DOI:** 10.1038/s41598-021-01950-8

**Published:** 2021-11-23

**Authors:** J. M. Kiszałkiewicz, S. Majewski, W. J. Piotrowski, P. Górski, D. Pastuszak-Lewandoska, M. Migdalska-Sęk, E. Brzeziańska-Lasota

**Affiliations:** 1 Department of Biomedicine and Genetics, Chair of Biology and Medical Microbiology, Medical University of Lodz, St. Pomorska 251, 92-213 Lodz, Poland; 2 Department of Pneumology, Medical University of Lodz, St. Kopcińskiego 22, 90-153 Lodz, Poland; 3 Department of Microbiology and Laboratory Medical Immunology, Chair of Biology and Medical Microbiology, Medical University of Lodz, St. Pomorska 251, 92-213 Lodz, Poland

**Keywords:** Genetics, Biomarkers, Diseases, Molecular medicine

## Abstract

COPD has been regarded as a global epidemic due to an increase in pollution and tobacco exposure. Therefore, the study of molecular mechanism as the basis for modern therapy is important. The aim of the study was the assessment of gene expression levels, *IL-6*, *IL-6ST*, *PIAS3*, *STAT3*, and miRNAs, miRNA-1, miRNA-106b, miRNA-155, in patients with COPD. Induced sputum as well as PBMC were collected from 40 patients clinically verified according to the GOLD 2021 (A–D) classification and from the control group (n = 20). The levels of gene and miRNA expression were analysed by qPCR. In induced sputum *IL6* was significantly down-regulated in COPD group compared with control (*p* = 0.0008), while *IL6ST* were up-regulated (*p* = 0.05). The results were also statistically significant for *STAT3* (*p* = 0.04) and miRNA-155 (*p* = 0.03) with higher expression in the current smokers compared to ex-smokers. Higher expression levels for *IL6ST* (*p* = 0.03) in COPD patients with the exacerbation history compared to COPD patients without the exacerbation history were noted. Compared induced sputum and PB lymphocytes we observed higher expression of *IL6* (*p* = 0.0003)*, STAT3* (*p* = 0.000001) miRNA-106b (*p* = 0.000069 and miRNA-155 (*p* = 0.000016) in induced sputum with lower expression of *PIAS3* (*p* = 0.006), *IL6ST* (*p* = 0.002) and miRNA-1 (*p* = 0.001). Differences in gene expression levels of the IL-6/IL6ST/STAT3 pathway and miRNA depending on the smoking status and classification of patients according to GOLD suggest the importance of these genes in the pathogenesis of COPD and may indicate their potential utility in monitoring the course of the disease.

## Introduction

Chronic obstructive pulmonary disease (COPD) is a common and complex respiratory disease associated with chronic airway inflammation, leading to progressive airflow limitation and substantial extra-pulmonary systemic manifestations (1). COPD affects more than 5% of the population and is associated with high morbidity and mortality. Furthermore, the prevalence and burden of COPD are predicted to increase in the future, due to increased exposure to environmental risk factors (pollution, smoking), and the aging of the world’s population. Despite worldwide health care efforts, costs, and medical research, epidemiological data demonstrate a continuously increasing tendency in mortality of COPD patients. This observed tendency is contrary to the other reasons of key causes of people death, such as neoplasm, accidents, and cardiovascular diseases. COPD nowadays becoming the third leading cause of death in the world by 2020^1,2^. Because of its high prevalence and chronicity, COPD causes high frequent clinician office visits, frequent hospitalizations due to acute exacerbations, and the need for chronic therapy.

The etiopathology of COPD has not been fully understood, but it has been recognized that both genetic and environmental factors. So far, diagnosis of COPD is based on the confirmation of the presence of irreversible obstructive airflow limitation by means of spirometry. However, severity of obstruction assessed by the forced expiratory volume in 1 s (FEV_1_) does not directly reflect global burden and systemic manifestations of individual patients with COPD^3,4^. Actually, the goals of COPD assessment are to determine the level of airflow limitation, its impact on the patient’s health status (including symptoms) and the risk of future events (such as exacerbations, hospitalizations and death), in order to eventually guide the therapy^1,4^.

COPD pathophysiology involves inflammatory responses to inhaled harmful particles including cigarette smoke. This normal protective response to the inhaled toxins is amplified in COPD, leading to tissue destruction, impairment of the defense mechanisms that limit such destruction, and disruption of the repair mechanisms. In general, the inflammatory and structural changes in the airways increase with disease severity and persist even after smoking cessation. Besides inflammation, two other processes are involved in the pathogenesis of COPD—an imbalance between proteases and anti-proteases and an imbalance between oxidants and antioxidants (oxidative stress) in the lungs^5^.

Recently, an increasing interest in biological markers is observed, which could allow clinicians to assess various aspects of COPD and help them better evaluate a disease burden and risks in individual patients.

Due to the fact, that increasing concentration of the inflammatory mediators in COPD course may induce some systemic changes, the analysis of a chronic inflammatory process is of special importance as an analysis of a common pathogenic system of pulmonary and extrapulmonary lesions.

**Figure 1:**
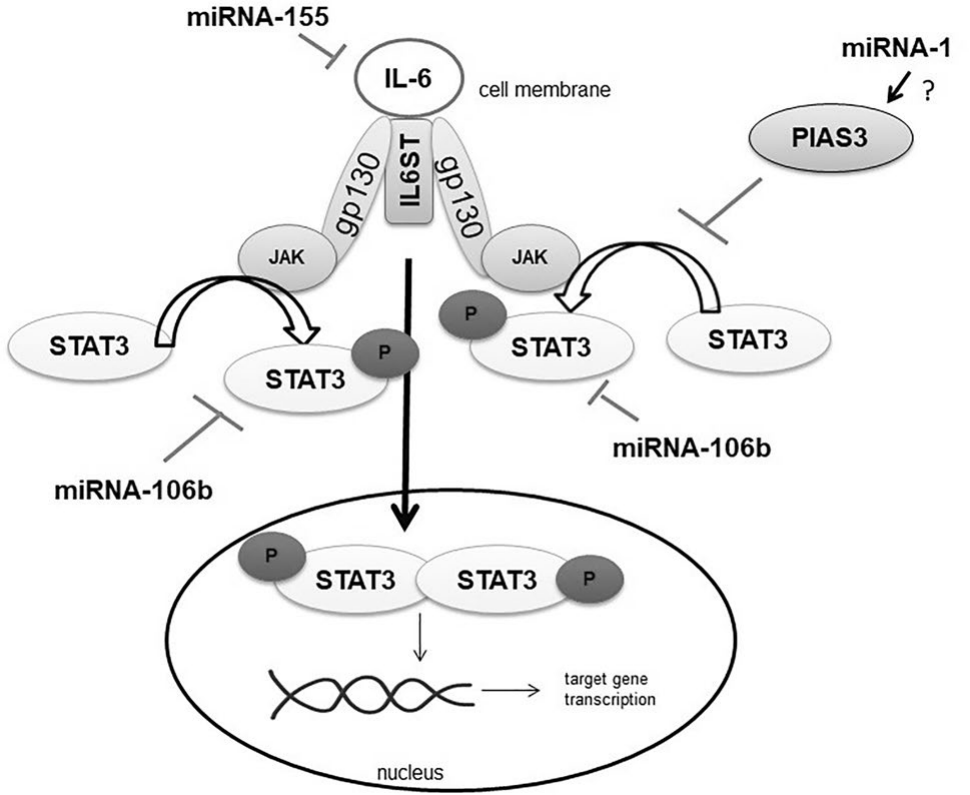
The IL-6/JAK/STAT3 signaling pathway. IL-6—interleukin 6. JAK—Janus kinase, IL6ST, gp130 (glycoprotein 130)—receptor for interleukin6, STAT3—signal transducer and activator of transcription 3, P—phosphorylation.

The strong evidence demonstrated that COPD is controlled by a complex set of interacting cytokines, mainly released by Th1, but there are reports about the importance of Th2 cells in the disease development (Th1/Th2 plasticity). It is known, that a number of pro-inflammatory cytokines, as important intracellular regulatory elements in global communication, may be associated with activation of IL-6/IL6ST/STAT3 signalling pathway, with recognized strong immunoregulatory function. Interestingly, inflammatory responses in the course of many diseases are determined by the binding of IL-6 to IL-6R via the activation of the JAK/STAT3 pathway. The signal path starts through the IL-6 receptor e.g. IL6R/IL6ST (GP130 a general receptor subunit for the interleukin-6 family of cytokines.) and then the activation of Janus kinases (JAKs). Before the activation, JAKs mediate the phosphorylation of the major transcriptional factor STAT3, which is regulated by IL-6^6,7^ (see Fig. 1). STAT3 (signal transducer and activator of transcription 3) is known to be an important regulator of inflammation, protease activation, and apoptosis processes which are involved in the pathogenesis of COPD and other autoimmune diseases like cancer or skeletal muscle function and disorders^8–13^.

It has been shown the IL6/IL6R/STAT3 signaling pathway influences neutrophil activity, which may be important in the pathogenesis of COPD. Additionally, secretion of IL-6, also independent of IL6/IL6R /STAT3, can affect the increase of neutrophil serine proteases (CTSG, PRTN3, Elan), which in addition contribute to the severity of inflammation and the destruction of lung tissue, as demonstrated in vitro and in an animal model^14,15^. The experiments on a mouse model demonstrated that the development of spontaneous emphysema is associated with excessive apoptosis and inflammation of the protease activity-dependent IL-6. At the molecular level, gp130 subunit mutation prevents inhibitor binding SOCS3 (suppressor of cytokine signaling), resulting in the activation of STAT3^16^.

The mechanism of endothelial cells and the stromal tissue inflammation is also regulated by intracellular protein PIAS (protein inhibitor of STAT), which inhibits the activation of cells, acting at different stages. PIAS proteins, constitutively present, may bind STAT proteins and block the gene transcription activities in vitro^17,18^, e.g. PIAS3 selectively inhibits STAT3 and expression of most genes induced by IL-6^17^.

Despite the published data confirming a significant role of the IL-6/IL6R/STAT3 signalling pathway in the inflammatory responses in different diseases in humans, little is known about its significance in COPD pathogenesis.

For several human genes, it has been demonstrated that their function is regulated via miRNAs^19^. miRNAs as small non-coding RNAs regulate the expression of many genes (nearly 30% of human genes) and may control almost all biological processes: cell growth, differentiation, apoptosis, adhesion, and cell death^20^. In addition, certain classes of miRNAs (e. g. miRNA-1, miRNA-106b miRNA-155, were recognized as involved in the modulation of the immune response and inflammation by inhibiting IL-6/STAT3 or PIAS3, a negative regulator of STAT3^21,22^. This choice was based on literature^21,22^ and also being awarded high target scores according to the miRDB database. MicroRNAs appear to be an important component in the differentiation of T cells and regulation of immune response. Recently, emerging data have been published confirming the importance of miRNA, e.g., miRNA-155 in the development and modulation of T-cell and dendritic cell function, or antigen-presenting cell (APC) population^22^.

The significance of our study is as follows: the proposed experimental study focused on molecular biology may improve the understanding of the complex molecular pathogenesis of COPD. The results concerning the expression levels of selected molecules i.e., STATs, their inhibitors PIAS and their regulators: miRNA, will allow the preliminary selection of new biological markers of COPD. The authors hypothesize that the results of the project will allow the verification of "panel" markers that are specific to the different stages of the disease. It may be possible that molecular biomarkers will possess prognostic value in the disease, especially in terms of predicting disease exacerbations. As so far a key challenge in COPD therapy is to create an agent that will limit inflammation without having a deleterious effect on innate immunity. To date, much progress has been made in the definition of cell-signalling pathways that are active during inflammation, and several targets have been proposed, particularly in immunological pathways. However, the targets for inhibition of STATs in macrophages in COPD are limited. The aim of this work is to search for genetic/epigenetic mechanisms that would be relevant in determining the IL-6/JAK/STAT3 signalling pathway in COPD. In our study, we assess gene and miRNAs expression in the PBMC and sputum of COPD patients. In addition, we analyze the relationship between the level of expression of selected genes and miRNA expression and clinical characteristics of COPD patients. At last, we did a comparative analysis of gene and miRNAs expression between induced sputum and PBMC.

## Materials

### Study population

Forty subjects, active or past smokers with a minimum of 10 pack-years history of smoking and diagnosed COPD, according to the Global Initiative for Chronic Obstructive Lung Disease guidelines (GOLD guidelines)^1^, were studied. All patients had to be in a stable condition defined as a disease without exacerbation for at least 1 month before study enrolment. Any other chronic respiratory condition than COPD, neoplasmatic disease or other coexisting significant chronic diseases (advanced heart failure, autoimmune, systemic or metabolic disease) were exclusion criteria. A control group consisted of 20 healthy active or past smokers with a minimum of 10 pack-years history of smoking and a normal spirometry. Control group subjects were free of any chronic respiratory conditions and free of a respiratory tract infection for at least 1 month prior to the study enrolment. This study was approved by the Ethics Committee of the Medical University of Lodz (RNN/105/14/KE) and was carried out according to the Declaration of Helsinki principles. All participants provided written informed consent before any study procedures were performed.

## Methods

All COPD subjects underwent clinical assessment including medical history, physical examination and symptoms evaluation using the modified Medical Research Council (mMRC) dyspnoea scale and COPD Assessment Test (CAT). Functional assessments included spirometry and 6 min walk test (6MWT). On the basis of available data, body mass index, airway obstruction, dyspnoea, and exercise capacity (BODE) index for each of the COPD subjects was also calculated^23^. To evaluate disease severity recruited COPD subjects were classified into four groups A–D according to the GOLD 2021 guidance^1^. All study subjects underwent the sputum induction procedure and blood draw sampling for further serum and sputum miRNA profiling.

### The mMRC dyspnea scale

mMRC is a five-level rating scale based on the patient’s perception of dyspnoea in daily activities. It consists of five statements that describe almost the entire range of dyspnoea from none (Grade 0) to almost complete incapacity (Grade 4)^24^.

### The CAT

The self-administered questionnaire consists of eight items assessing various manifestations of COPD aiming to provide a simple quantified measure of health related quality of life^25^. A decrease in CAT score represents an improvement in health status, whereas an increase in CAT score represents a worsening in health status. This questionnaire has been incorporated into the GOLD guidelines combined multidimensional assessment of specific patient attributes as a means of establishing a symptomatic threshold to guide stratification of the disease management^1^.

### Spirometry

Spirometry assessment was performed using a Lungtest 1000 spirometer (MES, Cracow, Poland) according to ATS/ERS guidelines^26^.

### The 6 MWT

In our study, 6 MWT was performed using the methodology specified by the Polish Respiratory Society guidelines^27^.

### BODE index

This multidimensional scoring system for COPD patients evaluates the body mass index (BMI), measurement of airflow obstruction (FEV_1_% predicted), dyspnoea score (grade in mMRC scale), and exercise capacity (distance covered in 6MWT)^23^.

### Exacerbation history

For our research, we applied the GOLD 2021 event-based definition of COPD exacerbation^1^. Exacerbations were classified as moderate if the worsening of the patient's respiratory symptoms required antibiotics and/or oral corticosteroids; or severe if the patient required hospitalization. Subjects who had at least 1 exacerbation (moderate or severe) in the last 12 months were considered as those with exacerbation history.

### Sputum induction

The sputum induction procedure was performed by a trained technician using the method described previously^28^. Briefly, after salbutamol pretreatment (400 µg), aerosols of hypertonic saline (3%, 4% and 5%) were each inhaled for 7 min via ultrasonic nebulizer (DeVilbiss Ultraneb 3000, Sunrise Medical Ltd, USA) with an output of 1 ml/min. Patients were asked to cough into a container after each cycle. The procedure was monitored by spirometry assessments at baseline and after each saline inhalation. If there was a fall in FEV1 of ≥ 20% versus baseline, the procedure was discontinued. Fall in FEV1 of 10–19% was an indication to continue the induction with the same concentration of saline.

### Sputum analysis

The sputum was selected from the expectorate and processed within 2 h as described previously^28,29^. Selected sputum plugs were dispersed using dithiothreitol (DTT), then the suspension was filtered and a total cell count of leukocytes and viability was assessed. Cytospins were stained with May-Grünwald-Giemsa. Differential cell counts were performed on 400 non-squamous cells.

### RNA isolation

RNA isolation was performed using a mirVana™ miRNA Isolation Kit (Life Technologies, Carlsbad, CA, USA), according to the manufacturer’s protocol. The quality and quantity of isolated RNA was assessed spectrophotometrically (Eppendorf BioPhotometrTM Plus, Eppendorf, Hamburg, Germany). The purity of total RNA was determined using RNA Nano Chips LabChipplates (ratio of 16S to 18S fraction) and RNA/miRNA was evaluated by automated electrophoresis using RNA Pico and RNA Nano Chips LabChipplates in an Agilent 2100 Bioanalyzer (Agilent Technologies, Santa Clara, CA).

### RNA expression analysis

cDNA was transcribed from 100 ng of total RNA, using a High-Capacity cDNA Reverse Transcription Kit (Applied Biosystems, Carlsbad, CA) in a total volume of 20 μl according to the manufacturer’s protocol. The RT reaction was performed in a personal thermocycler (Eppendorf, Hamburg, Germany). The relative expression analysis was performed in 7900HT Fast Real-Time PCR System (Applied Biosystems, Carlsbad, CA) using TaqMan probes for the studied genes: *IL-6* (Hs00174131_m1), *IL6ST* (Hs00174360_m1), *STAT3* (Hs00374280_m1), and *PIAS3* (Hs00966035_m1). The expression levels (RQ values) of the studied genes were calculated using the delta–delta CT method, with the adjustment to the β-actin expression level and in relation to the expression level of calibrator (Human Lung Total RNA Ambion®), for which RQ value was equal to 1.

### miRNA expression analysis

cDNA was transcribed from 10 ng of total RNA using a TaqMan® MicroRNA Reverse Transcription Kit (Applied Biosystems, Carlsbad, CA) in a total volume of 15 μl according to the manufacturer’s protocol. The relative expression of miRNAs was assessed by qPCR reactions using TaqMan probes for the studied miRNA (Applied Biosystems, Carlsbad, CA) according to the manufacturer’s protocol. The following microRNA probes were used for the study: hsa-miRNA-1 (UGGA AUGUAAAGAAGUAUGUA), hsa-miRNA-106b (UAAAGUGCUGACAGUGCAGAU), hsa-miRNA-155 ( UUAAUGCUAAUCGUGAUAGGGGU). The plates were placed in the Applied Biosystems 7900HT Fast Real-Time PCR System (Applied Biosystems, Carlsbad, CA) according to manufacturer’s protocol. The expression levels (RQ values) of the studied miRNAs were calculated using the delta–delta CT method, with the adjustment to the level of 001,973 U6 snRNA expression (endogenous control) and to the expression of the calibrator (Human Lung Total RNA Ambion®), for which RQ value was equal to 1.

### Statistical analysis

The Mann–Whitney *U*-test were used to assess the relative expression level (RQ) of the studied genes and miRNAs in induced sputum and PBMC in: (a) COPD patients versus controls, (b) patients with higher risk of exacerbation (C and D GOLD category) compared to patients with lower risk of exacerbation (A and B GOLD category) (c) patients with more symptoms (B and D GOLD category) compared to group of patients with less symptoms (A and C GOLD category); (d) group of current smokers compared to ex-smokers; (e) patients with the exacerbation history compared to COPD patients without the exacerbation history. The Neuman–Keuls multiple comparison test were used to assess the relative expression levels of the studied genes and miRNAs in induced sputum and PBMC in COPD patients according to GOLD classification, The Wilcoxon test were used to assess the relative expression levels of the studied genes and miRNAs, induced sputum vs. PB lymphocytes in COPD patients and Spearman’s rank correlation were used to assess the correlations. *p* = 0.05 was regarded as the level of statistical significance (StatSoft 13.1, Cracow, Poland).

### Ethical approval

The study was approved by the Ethics Committee of the Medical University of Lodz (RNN/105/14/KE). Written informed consent was obtained from all study participants before the study enrolment. All procedures performed in studies involving human participants were in accordance with the ethical standards of the institutional and/or national research committee and with the 1964 Helsinki declaration and its later amendments or comparable ethical standards.

## Results

### Characteristics of participants

The summary of characteristics of study participants is shown in Table 1. COPD patients were older than the healthy control group and had greater mean smoking exposure. The mean time since diagnosis of COPD was 6.66 ± 5.91 years and mean FEV_1_ was 60.95 ± 16.05% of the predicted value. COPD patients were symptomatic with a mean CAT score of 14.87 ± 7.41 points.

**Table 1:** Participants’ characteristics.

Characteristics	COPD	Control group	*p* value
Number of subject	40	20	N/A
Sex (male/female)	24/16	10/10	0.46
Age (years)	67.61 ± 6.88	57.73 ± 8.94	0.000041*
Time since diagnosis (years)	6.66 ± 5.91	N/A	N/A
Smoking exposure (pack-years)	42.25 ± 17.16	32.14 ± 12.57	0.027 *
Smoking status: never smokers/ex-smokers/current smokers n (%)	0 (0%)/23 (57.5%)/17 (42.5%)	0 (0%)/8 (40%)/12 (60%)	N/A
FEV_1_ (l)	1.51 ± 0.42	3.06 ± 0.65	0.000000*
FEV_1_ (% predicted)	60.95 ± 16.05	96.87 ± 26.56	0.000000*
FEV_1_/FVC	51.73 ± 9.61	74.39 ± 5.1	0.000000*
BMI (kg/m^2^)	28.24 ± 6.68	27.31	0.809
6MWT (m)	388.45 ± 69.15	N/A	N/A
CAT (score)	14.87 ± 7.41	N/A	N/A
mMRC (median, min–max)	1, 0–3	N/A	N/A
BODE index (median, min–max)	1.5, 0–6	N/A	N/A
Exacerbation history (with/without)	11/29	N/A	See point 4.5
Number of subjects according to GOLD classification (A/B/C/D)	10/21/2/7	N/A	N/A
**Inhaled COPD therapy (number of subjects on particular treatment)**			
LABA	5	N/A	N/A
LAMA	4	N/A	N/A
LABA/LAMA	17	N/A	N/A
LABA/LAMA/ICS	10	N/A	N/A
SABA	33	N/A	N/A
SAMA	7	N/A	N/A

*6MWT* minute walk test, *BMI* body mass index, *BODE* BMI, airway obstruction, dyspnoea, exercise capacity, *CAT* COPD assessment test, *COPD* chronic obstructive pulmonary disease, *FEV*_*1*_ forced expiratory volume in 1 s, *FVC* forced vital capacity, *mMRC* modified Medical Research Council dyspnoea scale, *ICS* inhaled corticosteroid, *LABA* long-acting β2-agonist, *LAMA* long-acting muscarinic antagonist, *SABA* short-acting β2-agonist, *N/A* not applicable.

*Statistically significant differences *p* < 0.005.

### Relative expression level (RQ) of the studied genes and miRNAs in COPD patients versus controls

#### Induced sputum

*IL6* mRNA was significantly down-regulated in COPD group compared with control group (*p* = 0.0008), while *IL6ST* mRNA were significantly up-regulated in COPD group compared with control group (*p* = 0.05). Higher gene expression levels for *PIAS3* and *STAT3* were observed in COPD patients compared to controls, but the difference did not reach the statistical significance. The expression of al studied miRNAs, although higher in the COPD group were not significantly different from the control group (Table 2).

**Table 2:** The expression levels (mean RQ value) of the studied genes and miRNAs in the COPD and control group in induced sputum.

	COPD group (mean RQ value ± SD)	Control group (mean RQ value)	*p* value
**General cohort**			
*Genes*			
*IL-6*	0.259 ± 0.697	0.304 ± 0.251	0.0008*
*IL6ST*	0.191 ± 0.204	0.113 ± 0.09	0.05*
*PIAS3*	0.554 ± 1.73	0.188 ± 0.100	0.19
*STAT3*	0.941 ± 1.065	0.758 ± 0.596	0.61
*miRNA*			
miRNA-1	0.531 ± 1.361	0.211 ± 0.450	0.99
miRNA-106b	11.21 ± 11.733	6.49 ± 6.08	0.15
miRNA-155	154.78 ± 380.54	94.29 ± 162.05	0.25
**Current smokers**			
*Genes*			
*IL-6*	0.442 ± 0.874	0.334 ± 0.293	0.12
*IL6ST*	0.163 ± 0.155	0.106 ± 0.08	0.39
*PIAS3*	0.288 ± 0.549	0.171 ± 0.075	0.11
*STAT3*	1.05 ± 0.850	0.607 ± 0.661	0.09
*miRNA*			
miRNA-1	0.649 ± 1.590	0.204 ± 0.522	0.42
miRNA-106b	8.826 ± 11.368	6.08 ± 5.34	0.87
miRNA-155	293.349 ± 542.342	147.195 ± 194.168	0.49
**Ex-smokers**			
*Genes*			
*IL-6*	0.187 ± 0.527	0.260 ± 0.180	0.008*
*IL6ST*	0.212 ± 0.234	0.123 ± 0.119	0.16
*PIAS3*	0.750 ± 2.23	0.214 ± 0.129	0.60
*STAT3*	0.859 ± 1.235	0.890 ± 0.494	0.35
*miRNA*			
miRNA-1	0.444 ± 1.195	0.222 ± 0.349	0.66
miRNA-106b	12.979 ± 11.934	7.11 ± 7.40	0.19
miRNA-155	52.322 ± 130.316	14.94 ± 6.46	0.06

*Statistically significant differences *p* < 0.005.

#### Peripheral blood (PB) lymphocytes

*STAT3* and *PIAS3* mRNA were significantly up-regulated in COPD group compared with control group (*p* = 0.000005, *p* = 0.0008 respectively), while miRNA-1 and miRNA-106b mRNA were significantly down-regulated in COPD group compared with control group (*p* = 0.000001, *p* = 0.000005 respectively). Higher gene expression levels for *IL6ST* and lower for *IL-6* were observed in COPD patients compared to controls, but the difference did not reach the statistical significance. The expression of miRNA-155, although lower in the COPD group were not significantly different from the control group (Table 3).

**Table 3:** The expression levels (mean RQ value) of the studied genes and miRNAs in COPD and control group in peripheral blood (PB) lymphocytes.

	COPD group (mean RQ value ± SD)	Control group (mean RQ value)	*p* value
**General cohort**			
*Genes*			
*IL-6*	0.007 ± 0.005	0.01 ± 0.015	0.22
*IL6ST*	0.497 ± 0.474	0.450 ± 0.525	0.20
*PIAS3*	0.518 ± 1.278	0.184 ± 0.080	0.0008 *
*STAT3*	0.257 ± 0.220	0.08 ± 0.044	0.000005 *
*miRNA*			
miRNA-1	0.631 ± 0.865	73.54 ± 106.54	0.000001 *
miRNA-106b	0.01 ± 0.017	92.24 ± 1555.90	0.000005 *
miRNA-155	1.64 ± 0.914	3.20 ± 6.694	0.24
**Current smokers**			
*Genes*			
*IL-6*	0.05 ± 0.005	0.012 ± 0.015	0.35
*IL6ST*	0.641 ± 0.614	0.324 ± 0.338	0.059
*PIAS3*	0.334 ± 0.149	0.176 ± 0.05	0.002*
*STAT3*	0.205 ± 0.111	0.078 ± 0.042	0.001*
*miRNA*			
miRNA-1	0.319 ± 0.266	72.255 ± 103.366	0.000042*
miRNA-106b	0.012 ± 0.006	128.340 ± 182.989	0.01*
miRNA-155	1.624 ± 0.851	4.624 ± 8.440	0.84
**Ex-smokers**			
*Genes*			
*IL-6*	0.008 ± 0.005	0.016 ± 0.015	0.20
*IL6ST*	0.391 ± 0.310	0.639 ± 0.707	0.85
*PIAS3*	0.654 ± 1.684	0.196 ± 0.113	0.09
*STAT3*	0.295 ± 0.271	0.084 ± 0.051	0.005*
*miRNA*			
miRNA-1	0.862 ± 1.069	75.487 ± 118.373	0.02*
miRNA-106b	0.021 ± 0.207	20.044 ± 23.796	0.009*
miRNA-155	1.666 ± 0.977	1.073 ± 1.030	0.09

*Statistically significant differences *p* < 0.005.

Also in current smokers we found statistically significant higher expression of *PIAS3* (*p* = 0.002), and *STAT3* (*p* = 0.001) in COPD patients and miRNA-1(*p* = 0.000042), miRNA106b (*p* = 0.01) in control group. In group of ex-smokers we observed statistically significant higher expression of *STAT3* (*p* = 0.005) in COPD patients and miRNA-1 (*p* = 0.02), miRNA106b (*p* = 0.009) in control group.

### Relative expression levels of the studied genes and miRNAs in COPD patients according to GOLD classification

#### Induced sputum

For all studied genes and miRNAs, there were no statistically significant differences between groups A–D according to the GOLD classification (*p* > 0.05). In Figs. S.1 and S.2 in Supplement is presented mean RQ of each gene and miRNA according to GOLD classification.

**Figure 2:**
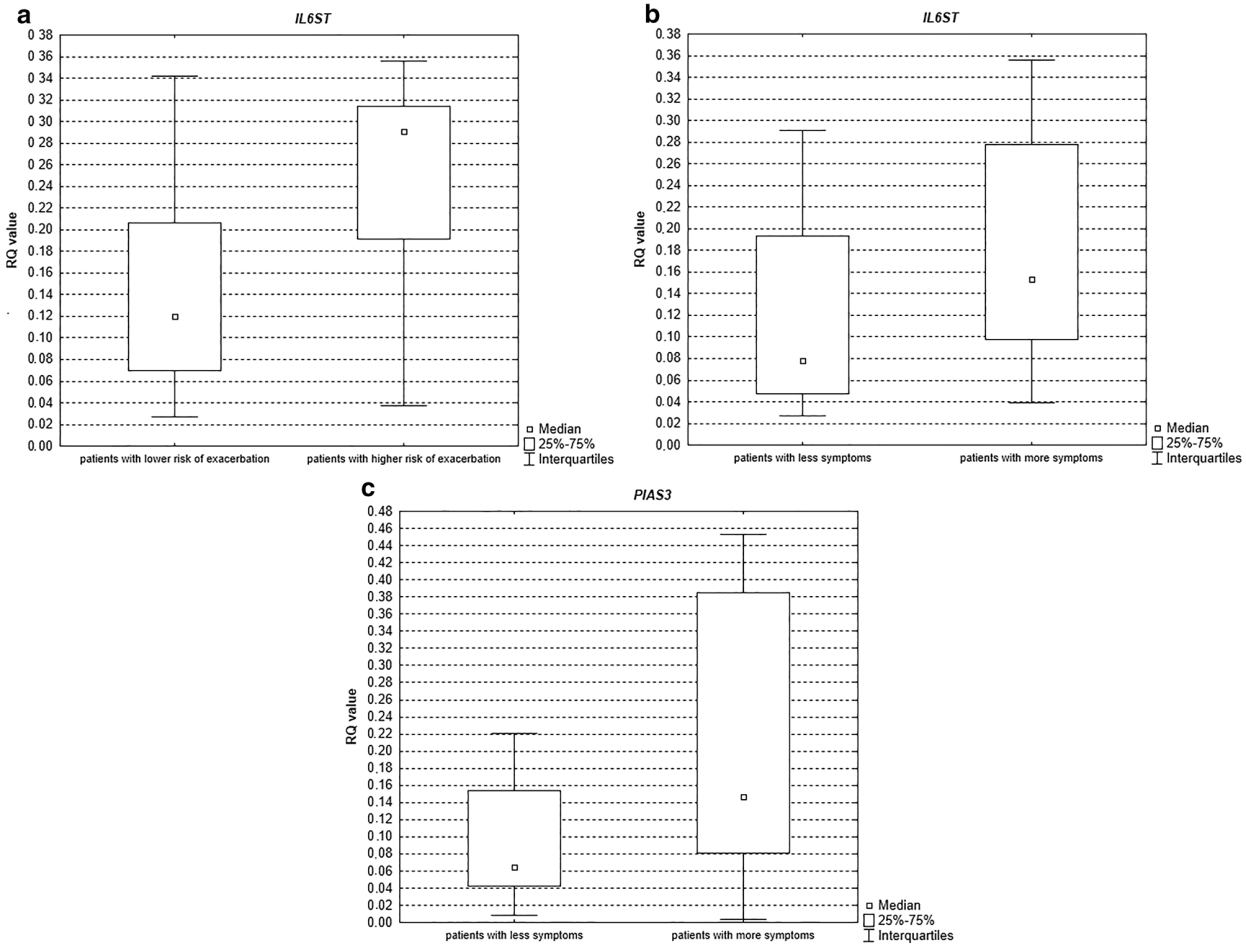
Box-and-whisker plots representing the expression value (median RQ value) of the studied genes in induced sputum: (**a**) *IL6ST* (*p* = 0.01) (**b**) *IL6ST* (*p* = 0.04) (**c**) *PIAS3* (*p* = 0.04).

However, there were statistically significant differences for *IL-6ST* patients with higher risk of exacerbation (C and D GOLD category) compared to patients with lower risk of exacerbation (A and B GOLD category) (*p* = 0.01, see Fig. 2a) and in group of patients with more symptoms (B and D GOLD category) compared to group of patients with less symptoms (A and C GOLD category) (*p* = 0.04, see Fig. 2b). For *PIAS3,* statistically significant differences were detected in group of patients with more symptoms (B and D GOLD category) compared to group of patients with less symptoms (A and C GOLD category) (*p* = 0.04, see Fig. 2c).

#### Peripheral blood (PB) lymphocytes

For all studied genes and miRNAs, there were no statistically significant differences between groups A–D according to the GOLD classification (*p* > 0.05). In Figs. S.3 and S.4 in Supplement is presented mean RQ of each gene and miRNA according to GOLD classification.

### Relative expression levels of the studied genes and miRNAs in COPD patients according to smoking status

**Figure 3:**
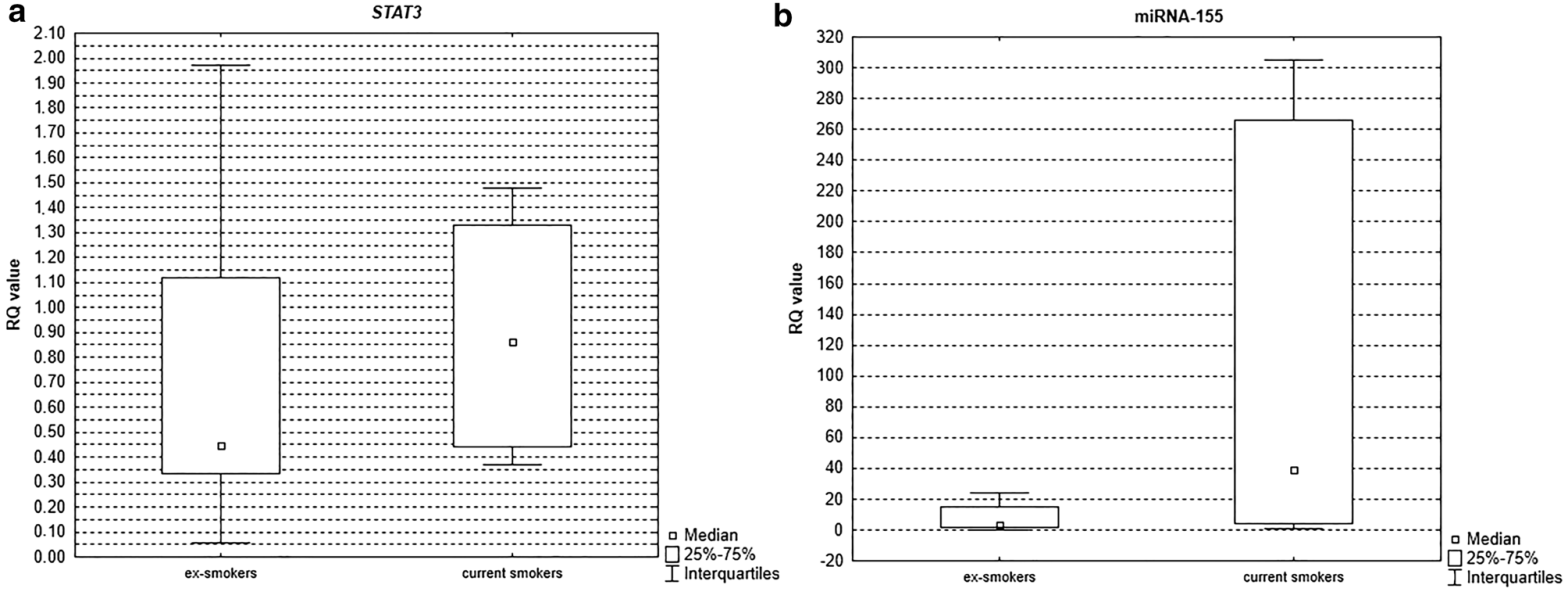
Box-and-whisker plots representing the expression value (median RQ value) of (**a**) *STAT3* (*p* = 0.04) (**b**) miRNA-155 (*p* = 0.03) in COPD patients according to the actual smoking status in induced sputum.

Analysis of miRNA and gene expression levels in induced sputum revealed statistically significant differences between the group of current smokers compared to ex-smokers for gene *STAT3* (*p* = 0.04, see Fig. 3a) and miRNA-155 (*p* = 0.03, see Fig. 3b) with higher levels of expression noted in the group of current smokers. There were no statistically significant differences between these groups for PB lymphocytes (*p* > 0.05).

### Relative expression levels of the studied genes and miRNAs in COPD patients according to the exacerbation history

**Figure 4:**
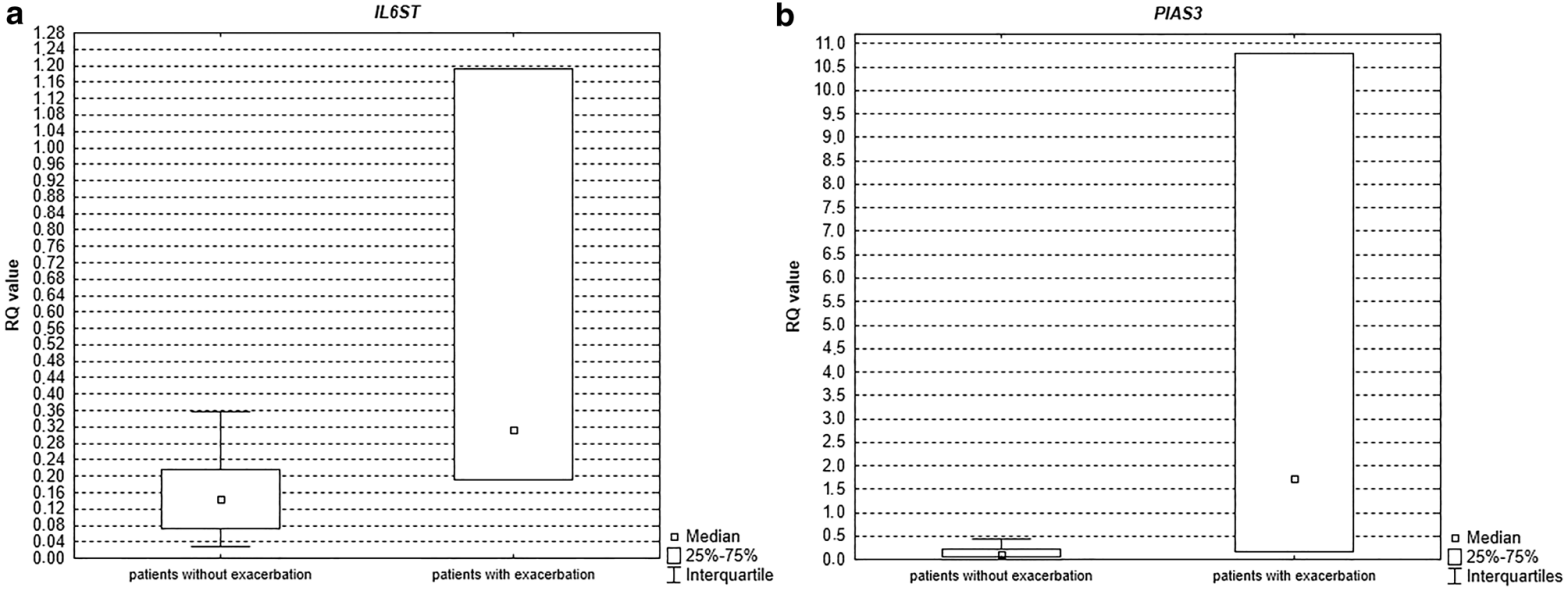
Box-and-whisker plots representing the expression value (median RQ value) of (**a**) *IL6ST* (*p* = 0.03) (**b**) *PIAS3* (*p* = 0.02) in COPD patients according to the exacerbation history in induced sputum.

We observed statistically higher expression levels for *IL6ST* (*p* = 0.03, see Fig. 4a) and *PIAS3* (*p* = 0.02, see Fig. 4b), in induced sputum in COPD patients with the exacerbation history compared to COPD patients without the exacerbation history in last 12 months. The mean expression levels of the studied genes and miRNAs in COPD patients with/without the exacerbation history is shown in Table 4.

**Table 4:** The expression levels (mean RQ value) of the studied genes and miRNAs in COPD patients with/without the exacerbation history.

	Exacerbation history	Without the exacerbation history in last 12 months	*p* value
**Induced sputum**			
*Genes*			
*IL-6*	0.393 ± 0.667	0.287 ± 0.708	0.96
*IL6ST*	0.565 ± 0.545	0.161 ± 0.126	0.03*
*PIAS3*	4.230 ± 5.737	0.255 ± 0.458	0.02
*STAT3*	0.505 ± 0.310	0.976 ± 1.099	0.19
*miRNA*			
miRNA-1	0.019 ± 0.014	0.573 ± 1.408	0.55
miRNA-106b	15.892 ± 10.020	10.835 ± 11.899	0.27
miRNA-155	9.959 ± 6.933	166.499 ± 393.684	0.1
**Peripheral blood (PB) lymphocytes**			
*Genes*			
*IL-6*	0.007 ± 0.004	0.007 ± 0.005	0.62
*IL6ST*	0.163 ± 0.132	0.525 ± 0.482	0.19
*PIAS3*	0.265 ± 0.180	0.539 ± 0.02	0.60
*STAT3*	0.191 ± 0.103	0.262 ± 0.227	0.64
*miRNA*			
miRNA-1	0.561 ± 0.034	0.637 ± 0.900	0.32
miRNA-106b	0.01 ± 0.0008	0.019 ± 0.018	0.36
miRNA-155	1.738 ± 0.823	1.641 ± 0.931	0.64

### Relative expression levels of the studied genes and miRNAs, induced sputum versus PB lymphocytes in COPD patients

**Figure 5:**
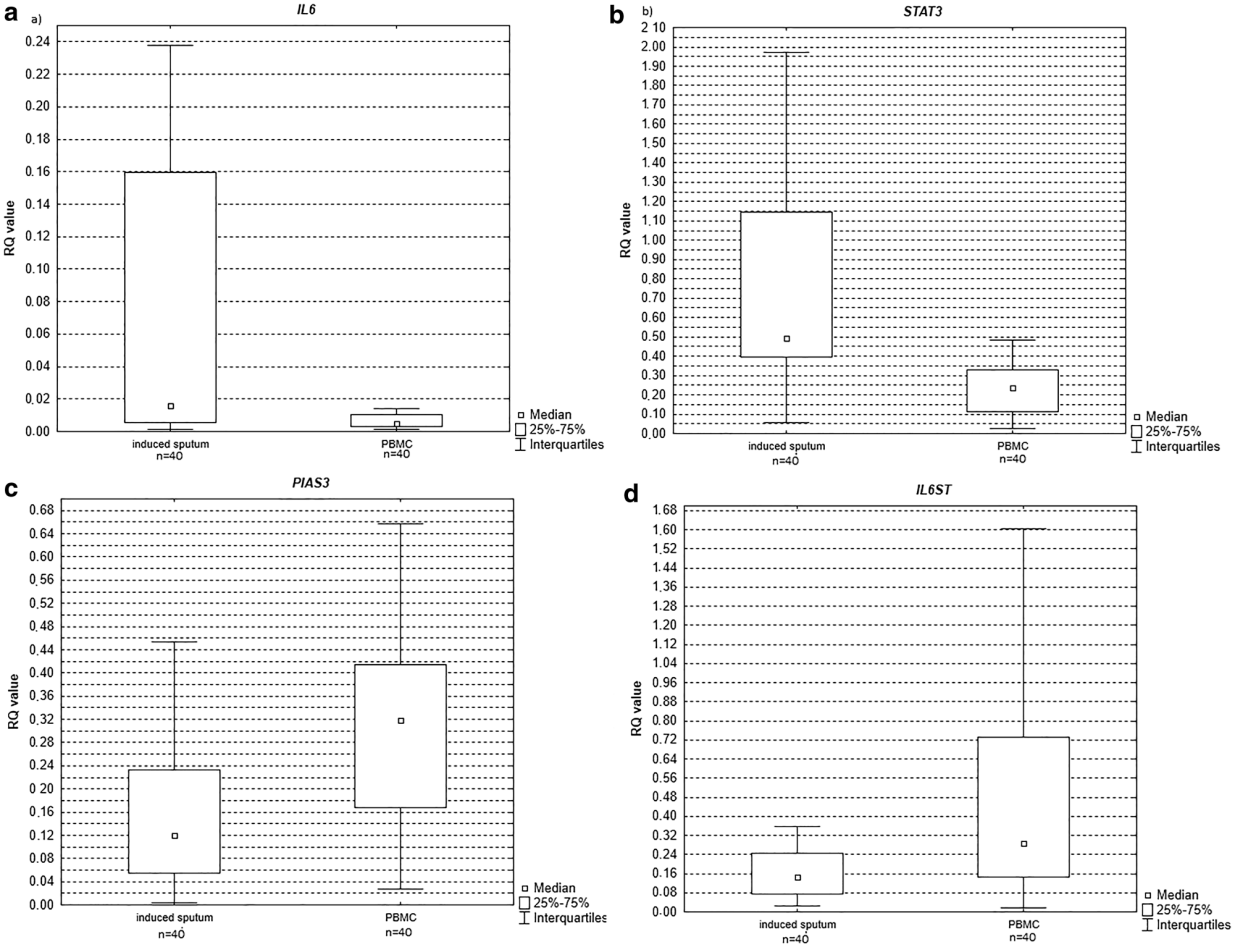
Box-and-whisker plots representing the expression value (median RQ value) of (**a**) *IL-6* (*p* = 0.0003) (**b**) *STAT3* (*p* = 0.000001) (**c**) *PIAS3* (*p* = 0.006) (**d**) *IL6ST* (*p* = 0.002) in induced sputum versus PB lymphocytes in COPD patients.

Statistically significant differences in expression levels between induced sputum and PB lymphocytes were observed for all studied genes with higher expression level of *IL6* (*p* = 0.0003see Fig. 5a)*, STAT3* (*p* = 0.000001 see Fig. 5b) noted in induced sputum and lower for *PIAS3* (*p* = 0.006 see Fig. 5c) and *IL6ST* (*p* = 0.002 see Fig. 5d).

**Figure 6:**
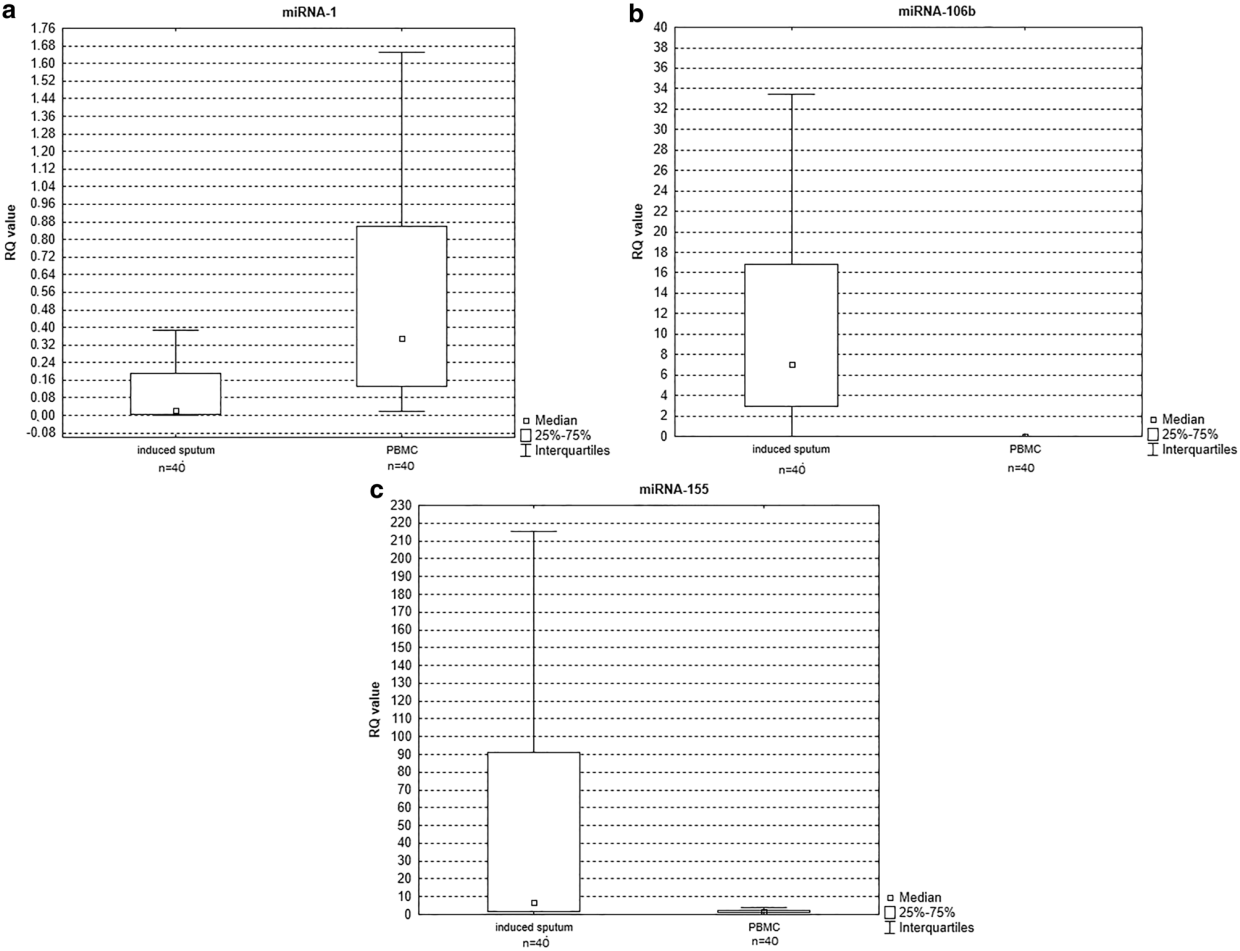
Box-and-whisker plots representing the expression value (median RQ value) of (**a**) miRNA-1 (*p* = 0.001) (**b**) miRNA-106b (*p* = 0.000069) (**c**) miRNA-155 (*p* = 0.000016) in induced sputum versus PB lymphocytes in COPD patients.

None of the studied miRNAs demonstrated overlapping patterns in induced sputum and PB lymphocytes. Statistically significant differences in expression levels between induced sputum and PB lymphocytes were observed for miRNA-106b (*p* = 0.000069, see Fig. 6b) and miRNA-155 (*p* = 0.000016, see Fig. 6c) with a higher expression level noted in induced sputum, and for miRNA-1 (*p* = 0.001, see Fig. 6a.) with a higher expression level in PB lymphocytes.

## Discussion

In our study, selected IL-6/STAT3 pathway molecules (e.g. IL-6, PIAS3, STAT3) on their mRNA level and miRNAs that can regulate mentioned above gene expression, have been assessed in peripheral blood lymphocytes and induced sputum leukocytes in patients with stable COPD.

IL-6 has pleiotropic properties: pro-inflammatory mediator and acute-phase response inducer and anti-inflammatory cytokine with major inhibitory effects on tumor necrosis factor-α (TNF-α) and IL-1^30^. IL-6 can contribute to tissue destruction by deposition of the matrix, antibody complexes, and proteases in the targeted tissue^31^. According to the earlier published data, IL-6 acts in COPD as a pro-inflammatory cytokine, and its secretion is also related to epithelial apoptosis and injury^32^. The increased levels of IL-6 were reported in sputum^33,34^, exhaled breath condensate^35^, blood^36^, and primary human fibroblasts in COPD patients^37^. Contrary to the above-mentioned data in our study, we noted a lower expression of IL-6 in COPD patients compared to the control group, while the IL-6ST expression level was higher in COPD patients in blood and sputum. However, studies of some authors reported consistent results with ours. For example, Harting et al.^38^ suggested that COPD patients contained lower levels of IL-6. Also, Moermans et al.^39^, found a lower expression level of IL-6 in cells from sputum in COPD patients compared to the control group. What are more, the findings of Foschino et al.^40^ and Liang et al.^6^ indicated that IL-6 mRNA expression levels in patients with stable COPD were only slightly higher compared to those in healthy controls. In our study we also observed a higher expression level of IL-6 in induced sputum in COPD patients with the exacerbation history (moderate ambulatory exacerbations requiring treatment with systemic steroids and/or antibiotics or severe exacerbations requiring hospital admission in last 12 months) compared to COPD patients without the exacerbation history. Our observation is consistent with the result of Bhowmik et al.^41^. They also observed higher IL-6 levels during an exacerbation when compared to the stable COPD phase^41^.

The unequivocal association of significantly increasing IL-6 inflammation among current smokers has been proven by others^42^. It is worth mentioning that in our study, many control subjects were active smokers. Therefore it is possible that their smoking status contributed to the higher expression of IL-6 in the control group observed by us in the study. Additionally, we confirmed that IL-6 levels were lower in former smokers compared to current smokers in a group of COPD patients. Unfortunately, we have not included non-smoking controls in our study, which could be useful to evaluate separately the possible effects of smoking and COPD on the expression of the studied molecules and miRNAs. On the other hand, the equivocal relationship of clinical outcomes with an increased IL-6 level suggests that IL-6 in the lung is not essentially a parameter of exacerbated inflammation, but rather a suggestive marker of a potential lung epithelium damage^43^. Thus, IL-6 has been attributed to the proinflammatory activities in COPD. However, it is unclear if this association is due to inflammation or whether IL-6 may come from damaged lung epithelial cells without the need for an exuberant inflammatory response in the lung. It may also happen that this process is modulated by other molecules (e.g. miRNAs). Indeed, in lung tissue and blood from COPD individuals, several cytokines such as IL-6 or TNF-a are found downregulated by the action of e. g miRNA-203^44^. It should be stressed that IL-6, as an activator of the JAK-STAT signaling pathway, can act as either pro-inflammatory as well as an anti-inflammatory factors in autoimmune and/or inflammatory diseases, including lung diseases.

Inhaled corticosteroids are anti-inflammatory medications that can modulate the systemic immune response of stable COPD patients. It is of note, that inhaled corticosteroids therapy may attenuate IL-6 expression in patients with COPD^45^ but the role of inhaled corticosteroids during exacerbations of COPD is still controversial. Surprisingly, we have observed the highest levels of IL-6 expression in group A of the COPD patients according to the GOLD classification, therefore in subjects with fewer symptoms and low risk of future exacerbations. Decreased levels of IL-6 expression could be an effect of inhaled corticosteroid use for the maintenance of disease treatment. Although, in our study, only 10 out of 40 COPD subjects were using ICS in the regimen of triple therapy LAM/LABA/ICS (5 patients belonged to group D, 4 patients were classified in group B and 1 patient was in group A). Therefore, ICS therapy could have influenced IL-6 levels in group D but not C, and not likely in group A or B as the majority of those patients were not using ICS. However, one should remember that noted differences in the genes and miRNAs expression levels between groups A–D in the PBMCs and induced sputum cells in our study were not statistically significant. In addition, the small sample size studied could have affected our study’s results and might lead to either under or overestimation of the observed effects. What is more, our study is consistent with results of Crisafulli et al.^46^. The author did not prove the differences in IL-6 level between subjects using or not using corticosteroids^46^. Moreover, Moreams et al.^39^ also confirmed that taking inhaled corticosteroids (ICS) did not seem to change the cytokine release from sputum or blood cell culture as there was no difference between those who were or were not on ICS^39^.

The specific role of STAT3 in the development of airway diseases such as COPD is still not clear. In normal macrophages, inflammatory responses are generated when STAT-3 is stimulated by the binding of IL-6^47^. Few papers are describing the effect of smoking on the level of STAT3 and the development of inflammation^48^. In our study, we found a significant increased of STAT3 expression in COPD current smokers compared to COPD ex-smokers. Qu et al.^49^ also found the upregulation of the STAT3 gene expression in COPD smoking patients. The same authors noticed that most of the genes regulated by STAT3 showed a modest increase in lung tissues of COPD smokers. Increased levels of STAT3 may have a prediction value in COPD patients. The degree of upregulation of STAT3 and its downstream genes may serve as an inflammatory warning sign for a pre-cancerous condition in the lung^49^. But is reported that the IL-6/STAT3 signalling pathway can have other properties. In Gp 130 Y757F mutant macrophages, the induction of transcriptional repressor can inhibit inflammatory gene responses in STAT-3 signaling by IL-6^50^. Therefore, efficient anti-inflammatory feedback can be stimulated with continuous Gp 130 and STAT-3 activation in SOCS3-deficient macrophages^51^.

According to the best of our knowledge, this is the first study assessing the PIAS3 mRNA expression level in COPD. It is likely that the PIAS3 mediated activity on multiple cell types and also been shown to suppress IL-6 production by bone marrow-derived macrophages and IL-10 production by NK cells and reverse IL-6–driven reduction in Treg-mediated suppression of T cells^52^. What is interesting in our study, we found higher expression levels of PIAS3 in induced sputum and peripheral blood lymphocytes in COPD patients compared to the control group. The highest PIAS3 expression level was higher in patients with more symptoms compared to those with less symptoms as well as between patients with exacerbation history compared to patients without exacerbation history in the last 12 months. These findings may suggest that PIAS3 can be a negative prognostic marker in COPD. To confirm this hypothesis, further studies in larger cohorts of COPD patients are required.

It is well known that miRNAs act as molecular regulators of various processes, signaling pathways, and different organ development including lungs. Several miRNAs, such as miRNA-16, miRNA-26a, miRNA-29, miRNA-17–92, miRNA-146, and miRNA-155 are thought to play an important role in the embryonic development of the lungs^53^. Among them, miRNA-155 is described also as a component of the primary response to inflammation. MiRNA-155 has an important role in the differentiation of T cells (Th1 and Th2)^54^. Wu et al.^55^ noticed that the stimulation of peripheral blood mononuclear cells (PBMCs) from active tuberculosis patients with purified protein derivative (PPD) leads to the activation of miRNA-155, suggesting a global role of miRNA-155 in the immune response in tuberculosis, which deserves further investigation. Another study by Kumar et al.^56^ showed a link between miRNA-155 and the innate immune response based on its increased expression following the stimulation of macrophages or monocytes with lipopolysaccharide (LPS) or lipoprotein. In our study, we focused on miRNA-155, as well as on miRNA-1 and miRNA-106b. We recorded an increased expression of all selected miRNAs in induced sputum cells of COPD patients, but a statistically significant higher level in COPD compared to the control group was observed only for miRNA-155. Kumar et al.^56^ concluded their findings that miRNA-155 inhibits the expression of the pro-inflammatory mediators e.g. IL-6. Therefore, it is plausible that the low expression of mRNA IL-6 observed in our study stems from the upregulated expression of miRNA-155. This finding suggests a regulatory role of miRNA-155 in COPD. However, the confirmation of this hypothesis requires further research.

In our study, we noted a significant overexpression of miRNA-155 in induced sputum of COPD current smokers compared to COPD ex-smokers. This finding may confirm the upregulation of miRNA-155 caused by active smoking.Ambient stimuli significantly affect the processes of transcription, translation, and expression of miRNAs. Takahasi et al.^57^ found changes in levels of smoking-induced serum expression of miRNAs. In a study by Soeda et al.^58^, researchers showed that the plasma miR-106b levels in current smokers and ex-smokers with COPD were decreased significantly compared to healthy smokers, which may suggest the concept that progressive reduction in the plasma miRNA-106b level may reflect persistent and systemic changes even after the discontinuation of smoking in COPD patients. Furthermore, miR-106b-5p might be potential markers of COPD. Wang et al.^59^ discover that miR-106b-5p was negatively correlated to the severity of COPD. Soeda et al.^58^ reported that miR-106b plasma levels are evidently downregulated in COPD and are negatively correlated with disease duration. What is compatible with our result, we also observed the lowest expression level of miRNA-106b from PBMC in patients with D category according to GOLD classification with the highest expression in the control group.

miR-1 has been linked to cigarette smoking-related conditions such as heart disease and cancer^60^. Is also highly expressed in skeletal muscle and can regulate muscle phenotype^61^. miR-1 promotes myotube formation. As so far only one study has described miRNA-1 in COPD^61^. In this study, miRNA-1 is down-regulated in skeletal muscle of patients with COPD compared with non-smoking controls, and its expression correlated with clinical features. In our study, we also found down-regulated miRNA-1 expression in PBMC in COPD patients compared to the control group. It is believed that that reduced physical activity commonly observed in patients with COPD would suppress SRF (serum response factor) activity and reduce expression of miR-1.

One of the most likely hypotheses of the genesis of systemic inflammation in COPD is the ‘spill-over of inflammatory mediators from the lung to the blood (reactive oxygen species and cytokines spilling over from the airways into the systemic circulation or peripheral liberation of pro^1^ inflammatory cytokines by inflammatory and/or structural cells), suggesting an association between these inflammatory processes^62^. Agusti et al.^63^ considered that the local inflammatory process in the lungs of COPD patients can affect peripheral tissues either directly by the release of cytokines and chemokines or indirectly by activation of peripheral inflammatory cells. However, the relationship between pulmonary and systemic inflammation in COPD is controversial and has yet to be identified.

The study of Núñez et al.^64^ shows the lack of correlation between different inflammatory markers determined simultaneously in blood and sputum in the same stable COPD patient. Nor were any significant associations found between these markers and lung function variables, such as FEV1, DLCO, and PaO2, or with age.

In our study, we noticed a statistically higher expression level of IL-6 and STAT3 and miRNA-106b, miRNA-155 in induced sputum compared to PBMC. Similar results were obtained by He et al.^65^. They suggest differential regulation of the inflammatory responses locally and systemically in stable COPD patients. They observed increases in inflammatory cell numbers, IL-6, and CRP levels occur earlier in the airway than in peripheral blood. Airway inflammation reflects the degree of airflow limitation better than inflammatory markers in peripheral blood. Also, Vernooy et al.^66^ studied the relationship between local and systemic inflammation in patients with mild to moderate smoking-related COPD. They suggested that the inflammatory responses in the local and systemic compartments were regulated differently. Takabatake et al.^67^ reported that the systemic hypoxia observed in patients with COPD might contribute to enhanced levels of systemic inflammatory markers. In our study, we observed a higher expression level of PIAS3 (PIAS3 overexpression enhanced the transcriptional activity of HIF-1α) in PBMC.

Some studies^68,69^ have shown that polymorphisms (especially in the TNF family, IL-6, and CRP genes) within genes coding for these inflammatory mediators may modulate systemic inflammatory responses. Also, more than one pathway may be operational at one time. Resuming all these data lung inflammation and systemic inflammation in patients with stable COPD do not occur concurrently and systemic inflammation may present in severe and very severe stable COPD patients. All these mechanisms may be involved in the different timing seen for lung and systemic inflammation in COPD.

## Study limitations

This study has several limitations, the most important being a small sample size studied and a small number of genes and miRNA evaluated which may reduce the statistical power of the experiment. Only a small fraction of the genome is examined during such an experiment. While one miRNA can regulate about 200 genes, to study in depth all the mechanisms under regulation, a larger number of genes and miRNAs are needed to be analysed. Differences in the age and smoking history between COPD patients and controls could also have influenced the obtained results. Taking into account the significant effect of cigarette smoking on the expression of the studied molecules makes it a potential limitation of the study. Another limitation is the lack of inclusion of non-smoking controls which could be useful to evaluate separately the effects of smoking and COPD on the expression of the studied molecules and miRNAs. Despite the fact, that the present explorative study has several limitations, in our opinion, the novelty of findings extends our current knowledge on the pathophysiologic pathways in COPD.

## Conclusions

The present study data showed overexpression of PIAS3, IL-6ST, STAT3, and miRNA-155 in COPD subjects as compared with the controls. These findings may suggest their significant roles in the disease-related inflammatory responses in the pathobiology of COPD. In addition, for the first time, we have demonstrated that PIAS3 may be a potential candidate for an adverse prognostic marker in COPD. Further studies in larger patients cohorts are warranted to validate our research exploratory findings.

## Supplementary Information

Supplementary Legends.

Supplementary Figure S1.

Supplementary Figure S2.

Supplementary Figure S3.

Supplementary Figure S4.
