# Supplementary material for: Evaluation of selected IL6/STAT3 pathway molecules and miRNA expression in chronic obstructive pulmonary disease
Source: Sci Rep. 2021 Nov 23;11:22756. doi: 10.1038/s41598-021-01950-8 (PMC8610981; doi:10.1038/s41598-021-01950-8)
Supplement: Supplementary file 1 — Supplementary Legends. [file 41598_2021_1950_MOESM1_ESM.doc]

**Fig. S.1** Relative expression levels (mean RQ value) of the studied genes, a) *IL-6,* b) *IL6ST,* c) *STAT3,* d) *PIAS3*, in COPD patients according to the GOLD classification and control group in induced sputum.

**Fig. S.2** Relative expression levels (mean RQ value) of the studied miRNAs, a) miRNA-1, b) miRNA-106b, c) miRNA-155, in COPD patients according to the GOLD classification and control group in induced sputum.

**Fig. S.3.** Relative expression levels (mean RQ value) of the studied genes, a) *IL-6,* b) *IL6ST,* c) *STAT3,* d) *PIAS3*, in COPD patients according to the GOLD and control group classification in peripheral blood (PB) lymphocytes

**Fig. S.4** Relative expression levels (mean RQ value) of the studied miRNAs, a) miRNA-1, b) miRNA-106b, c) miRNA-155, in COPD patients according to the GOLD classification and control group in peripheral blood (PB) lymphocytes
