# Supplementary figures and images for: Evaluation of selected IL6/STAT3 pathway molecules and miRNA expression in chronic obstructive pulmonary disease
Source: Sci Rep. 2021 Nov 23;11:22756. doi: 10.1038/s41598-021-01950-8 (PMC8610981; doi:10.1038/s41598-021-01950-8)

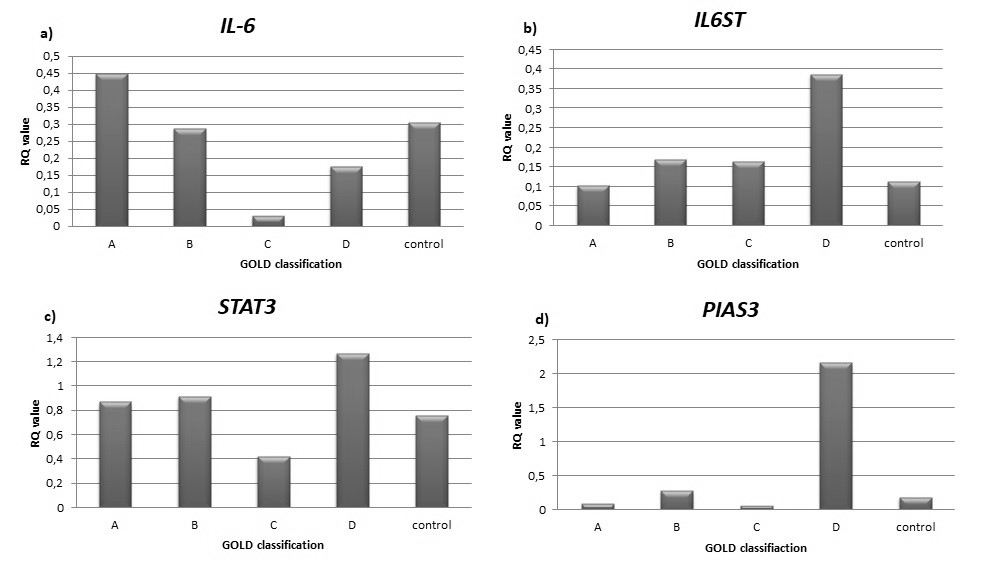

Supplement: Supplementary file 2 — Supplementary Figure S1. [file 41598_2021_1950_MOESM2_ESM.jpg]

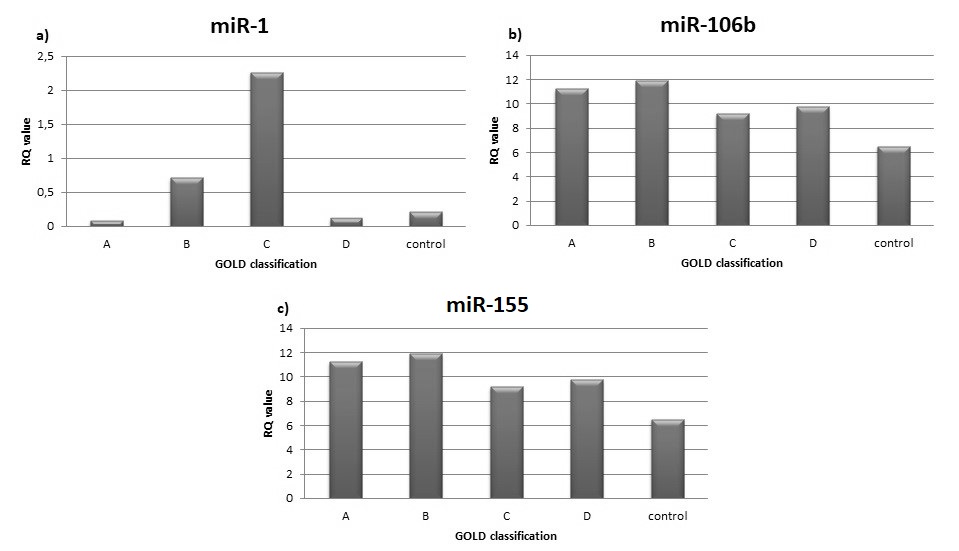

Supplement: Supplementary file 3 — Supplementary Figure S2. [file 41598_2021_1950_MOESM3_ESM.jpg]

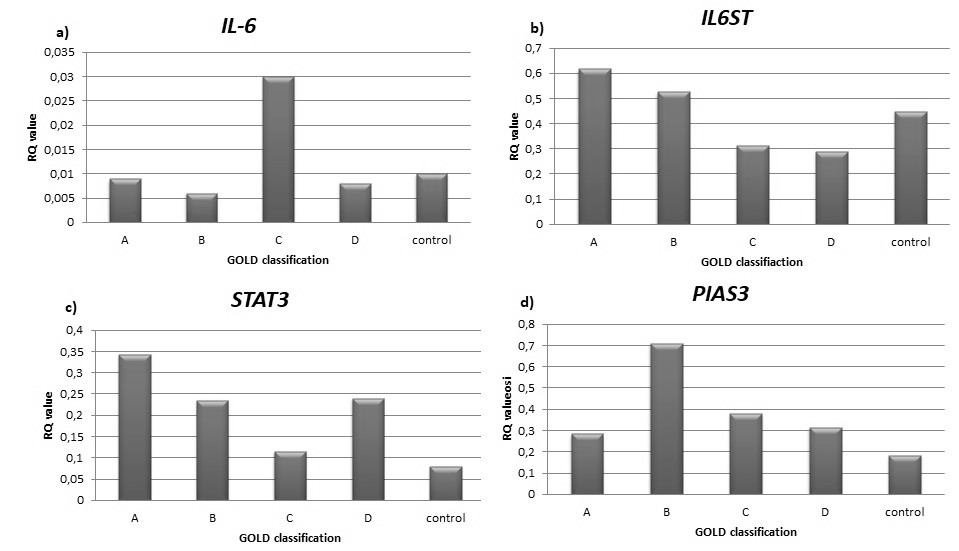

Supplement: Supplementary file 4 — Supplementary Figure S3. [file 41598_2021_1950_MOESM4_ESM.jpg]

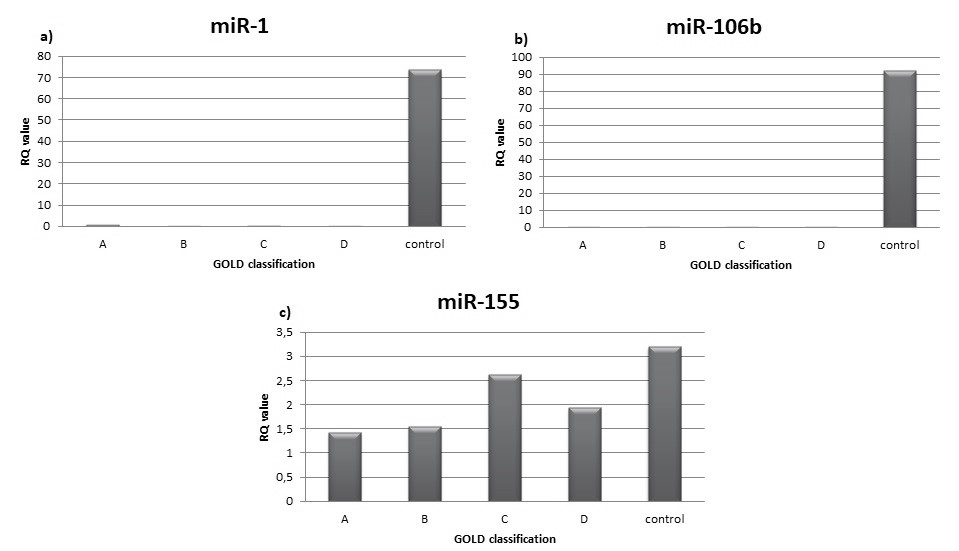

Supplement: Supplementary file 5 — Supplementary Figure S4. [file 41598_2021_1950_MOESM5_ESM.jpg]
